# Marginal resection of solitary plasmacytoma in the anterior region of the mandible and dental implant rehabilitation: Report of an unusual case

**DOI:** 10.1016/j.amsu.2018.10.028

**Published:** 2018-11-08

**Authors:** Katheleen Miranda dos Santos, Jennifer Tsi Gerber, Pedro Teruo Mendes Okazaki, Cibele Cândida de Almeida Kintopp, Leandro Eduardo Klüppel, Allan Fernando Giovanini, Rafaela Scariot

**Affiliations:** a Department of Oral and Maxillofacial Surgery, Universidade Positivo, Curitiba, Brazil; b Department of Dental Prosthesis, Universidade Positivo, Curitiba, Brazil; c Department of Oral and Maxillofacial Surgery, Universidade Federal do Paraná, Curitiba, Brazil; d Department of Oral Pathology, Universidade Positivo, Curitiba, Brazil

**Keywords:** Plasmacytoma, Jaw, Lesion, Rehabilitation, SP, solitary plasmacytoma

## Abstract

**Introduction:**

Plasmacytoma describes a neoplastic proliferation of plasma cells affecting different groups of patients according to location, and may demonstrate heterogeneous tumor progression and survival rate. The present article describes a case of solitary plasmacytoma in the mandible**.**

**Presentation of case:**

A 57-year-old male smoker was referred to the oral and maxillofacial service with extensive injury, approximately 4–5 cm in size, involving the anterior inferior region of mandible. After confirming diagnosis of plasmacytoma through incisional biopsy, because it was a recurrent lesion, excision of the lesion was performed through marginal resection of the mandible under general anesthesia. During the same surgical procedure, a 2.4-mm system fixation plate was placed to mitigate the risk for pathological fracture of the mandible. In a second surgery, a region in the base of the mandible was rehabilitated using implants and prosthesis.

**Conclusion:**

The patient is currently undergoing clinical and radiological follow-up of 2 years with success.

## Highlights

• Diagnosis and treatment of solitary plasmacytoma (SP) in the mandible is described.
• Marginal resection of the mandible and excision of the lesion proved effective.
• Rehabilitation with implants was chosen to provide stability and quality of life.
• Accurate diagnosis of SP is essential, and treatment varies in each case.

## 1 Introduction

Plasmacytoma is a pathological condition involving neoplastic proliferation of monoclonal plasma cells that commonly involves bone tissue [1,2]. This peculiar disease is usually classified according to its location and is termed solitary plasmacytoma (SP) when it has a single bone involvement, or multiple myeloma when it is polyostotic. Although rare, there is some evidence that may be found an extramedullary plasmacytoma when this pathological condition involvement exclusively affects the soft tissue [3]. It is noteworthy that when localized monoclonal proliferations of plasma cells occur, its progression to multifocal disseminated disease and multiple myeloma appears to be a common event―approximately 80% of cases―and, under these circumstances, the disease represents the most important, most severe and common plasma cell dyscrasia [4,5].

SPs of the bone usually arise in the vertebrae, ribs, pelvis and pectoral girdle [3]. Oral manifestations of SP include localized pain, paresthesia, swelling, soft tissue masses, mobility and migration of teeth, hemorrhage, and pathological fracture [6,7]. When present in the craniofacial bones, it exhibits radiographic characteristics represented by well-defined areas, with unilocular radiolucency or “punched-out” appearance, similar to multiple myeloma, to ill-defined destructive radiolucencies with ragged borders [8]. Radiation therapy, radical extensive surgery, or a combination of both, is recommended as primary treatment. Surgical treatment is recommended for situations in which the entire tumor must be removed to minimize esthetic or functional deficits, or in cases in which pathological fracture is anticipated [9]. The present case report describes the diagnosis and full treatment of SP in the mandible based on clinical, radiographic and histological characteristics.

## 2 Presentation of case

**Fig. 1:**
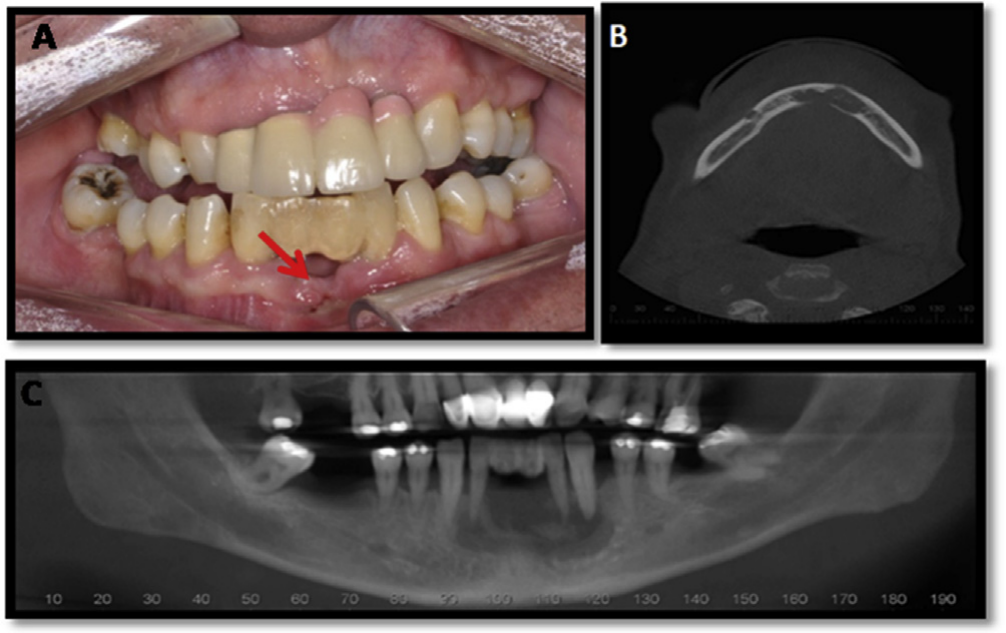
**A.** Intraoral image revealing initial clinical condition with drainage of purulent secretion. **B.** Axial computed tomography section revealing bucco-lingual extension of the lesion. **C.** Initial tomographic appearance of the lesion.

This report adheres to the SCARE Statement [10]. A 57-year-old male patient, smoker, and eventual alcoholic, was referred to the oral and maxillofacial surgery service for evaluation of a previous radiographic lesion found in the mandibular symphysis observed during a routine examination. There is no relevant psychosocial or family history. During anamnesis and physical examination, the patient reported pain in the anterior region of the mandible and spontaneous drainage of a purulent secretion. In addition, intraoral examination revealed unstable occlusion and precarious oral hygiene. The patient used a lower adhesive denture and exhibited a gingival deformity in the anterior region of the mandible (Fig. 1A). The patient perspective was only functional. After clinical examination, computed tomography was performed after an initial panoramic examination. In this imaging examination, an extensive unilocular radiolucent lesion was verified, measuring approximately 4–5 cm, with evident loss of cortical bone plate and resorption involving the inferior anterior teeth (Fig. 1B and C).

**Fig. 2:**
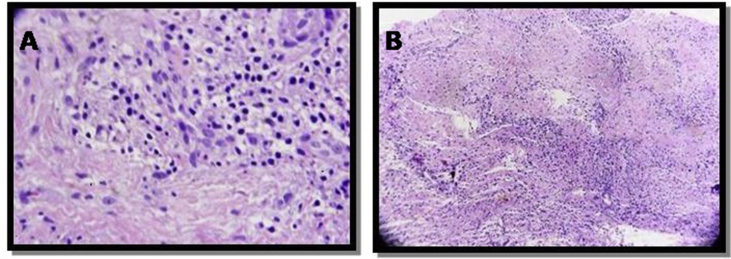
A and B. Histological appearance of the lesion.

Under local anesthesia, an incisional biopsy was performed by senior surgeons (LEK and RS) and a surgical fragment was sent for anatomopathological analysis. The surgical fragment exhibited a fibro-elastic consistency, with brownish coloration, measuring 4 × 3 × 3 mm. The fragment was hemisected and embedded in paraffin. Sections were obtained and stained with hematoxylin and eosin. In histopathological analysis, moderate to intense staining of plasma cells exhibiting eccentric nuclei and coarse chromatin condensed at the periphery was verified. These cells were permeated by fibrous and dense connective tissue. Thus, in view of the anatomopathological frame, a diagnosis of SP was established (Fig. 2A and B).

**Fig. 3:**
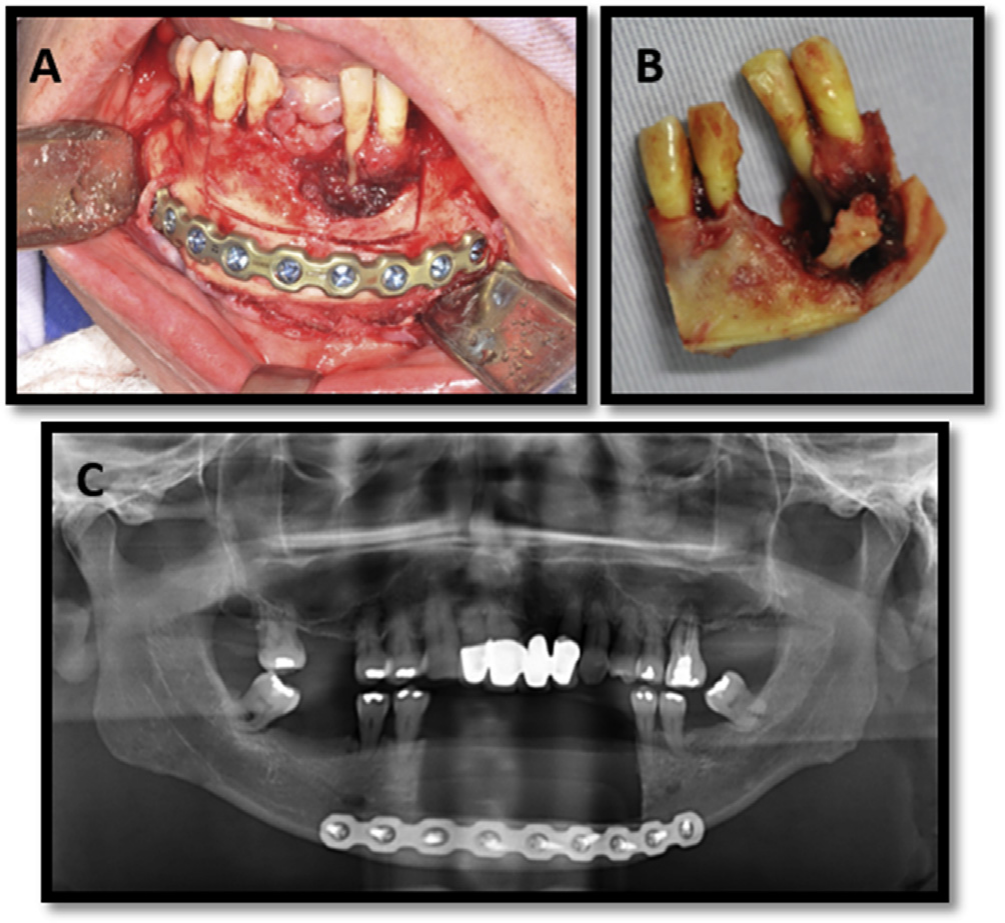
**A**. – Demarcation of the resected area and stabilization of the remaining bone using a 2.4-mm system fixation plate. **B.** Removed section. **C.** Post-surgery panoramic radiography confirming stability of the internal fixation.

**Fig. 4:**
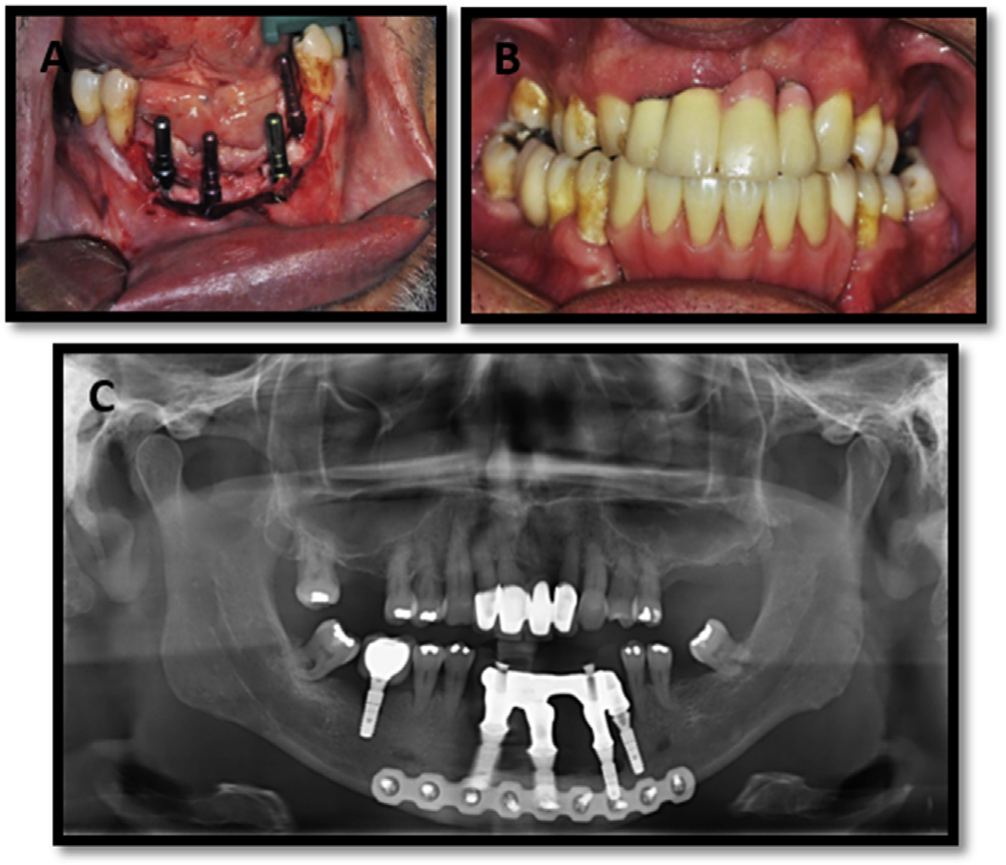
**A.** Rehabilitation with 4 implants, 8 months after resection surgery. **B** and **C.** Clinical and radiographic aspects after rehabilitation with implant-supported prosthesis.

The treatment plan involved marginal resection of the mandible for complete removal of the lesion with a safety margin under general anesthesia, performed by the same surgeons. Stable internal fixation in the base of the mandible was performed using reconstruction plates and 2.4-mm system screws to mitigate the risk for mandible fracture (Fig. 3 A and B). One week later, the patient underwent postoperative panoramic radiography, which confirmed removal of the lesion and satisfactory positioning of the plaque (Fig. 3 C). In this period, the patient continued use of a temporary removable prosthesis. Eight months post-surgery, the patient underwent rehabilitation with four implants (Straumann, Basel, Switzerland) in the base of the mandible (Fig. 4 A). Due to the height of the pillars and the distance between the bases of these pillars and the occlusal line, an acrylic “mini-protocol” was used to rehabilitate the patient. Acrylic was chosen so that it would not overwhelm the remaining bone (Fig. 4B and C). The patient was advised to sanitize the prosthesis with interdental brushes and devices that have air or water pressure. Follow up was performed every six months. The patient is undergoing 2 years of follow-up.

## 3 Discussion

Plasmacytoma is the result of uncontrolled monoclonal proliferation of B cells, without distant spread, which is capable of progressing to the stage of plasma cells [11]. SP is frequently diagnosed as a benign lesion, inflammatory disease or, less frequently, a malignant tumor. The most common clinical symptoms of SP are localized pain in the jaws and teeth, which could also be associated with other jaw lesions [12]. Moreover, paresthesia, swelling, mobility and migration of teeth, hemorrhage, and pathological fracture could also be clinical signs. Fatigue and fever are the most common systemic symptoms [6,7]. Canger et al. [12] reported that patients are usually male, with a male to female ratio of 2:1, and usually in their sixth or seventh decades in life. In the present case, the patient was a male in his late fifties.

SP represents approximately 3–10% of all plasma cell neoplasms [9]. An early diagnosis of solitary plasmacytoma of the bone is crucial to the survival rate of patients. The conversion to multiple myeloma occurs in approximately 70% of the cases, on average, 20.7 months after the initial diagnosis [13]. The mandible is a rare location for solitary plasmacytoma of the head and neck to arise [14]. Approximately 12%–15% of SPs occur in the jaw, and are commonly involved in the posterior body of the mandible, being able to extend to the angle and ramus [15]. In the present case, the lesion attacked the anterior region of mandible. The radiographic signs of SP include uni- or multilocular, well- or ill-defined radiolucent lesions, which may cause cortical bone expansion [12]. As a differential diagnosis, the most common lesions based in the clinical manifestation and radiographic characteristics include odontogenic myxoma, ameloblastoma and odontogenic keratocyst. In the present case, the initial diagnostic hypothesis was not plasmacytoma because of the absence of systemic signs and symptoms, as well as characteristics of the affected region. Initially, it was believed to be odontogenic keratocyst and odontogenic myxoma. Thus, the analysis of histopathological features is crucial to differentiate these pathological entities.

In microscopy, SP exhibits well-differentiated plasma cells, with small eccentric nuclei and granular chromatin condensed at the periphery. The tumors may also consist of immature plasma cells exhibiting finely dispersed nuclear chromatin in eccentric nuclei, prominent nucleoli and abundant cytoplasm [3]. Plasmacytomas usually occur due to clonal plasma cell proliferation that is cytologically and immunophenotypically similar to myeloma cells [13]. Considering only histological features, distinguishing between SP and myeloma can be complicated [16]. In these circumstances, it should be emphasized to the health professional that some systemic evidence should be considered when evaluating these patients. Among this evidence, normocytic and normochromic anemic syndrome, as well as hypercalcemia, is fundamental. An examination to verify ionic fraction or total calcium should be performed, especially in patients who exhibit signs of lethargy, polyuria, constipation, nausea, or vomiting. Furthermore, a renal biopsy to verify the presence of Bence-Jones protein, which is considered the gold standard to diagnose multiple myeloma, as well as hyperuricemia, hypercalciuria, and signs of dehydration should be part of routine practice.

Treatment of SP consists of surgery, radiotherapy or a combination of both. There is, however, some controversy in relation to the sole use of surgery or radiotherapy [17]. In a previous study, radical radiotherapy yielded 80% control of local disease. Nevertheless, a first surgery may be advantageous in relation to the exclusive use of radiotherapy, depending on the size and the location of the lesion [18]. In the present case, the patient was a smoker with an extensive lesion in the anterior region of the mandible. Because there were no systemic symptoms or signs of the lesion, surgical excision was performed without radiotherapy. Rehabilitation involving implants was chosen to provide stability and quality of life. We preferred not to perform a reconstruction using bone graft in the resected area due to the patient's harmful habits and the lack of cooperation, being this, one of the peculiar point of our approach. All patients with plasmacytoma require follow-up for at least 5 years after treatment has concluded. The course of SP in bone is relatively benign; its 5-year survival rate is 60%; however, it falls to 5.7% when progression to multiple lesions occurs [8].

## 4 Conclusion

Based on the above considerations, an accurate diagnosis of SP is essential, and treatment varies according to each case. In this study, marginal resection of the mandible and excision of the lesion proved to be an effective alternative treatment option.

## Ethical approval

There is no ethical approval because it is not research study. We have the written consent of patient to published this case report.

## Sources of funding for your research

None of the author.

## Conflicts of interest

None of the author.

## Guarantor

Rafaela Scariot.

## Consent

Written informed consent was obtained from the patient for publication of this case report and accompanying images.

## Registration of research studies

This paper is only case report.

## Provenance and peer review

Not commissioned, externally peer reviewed.

## Appendix A Supplementary data

SCARE Guideline Checklist SCARE Guideline Checklist

## References

[1] Matsumura S., Kishino M., Ishida T., Furukawa S. (2000). Radiographic findings for solitary plasmacytoma of the bone in the anterior wall of the maxillary sinus: a case report. Oral Surg. Oral Med. Oral Pathol. Oral Radiol. Endod..

[2] Pisano J., Coupland R., Chen S., Miller A. (1997). Plasmacytoma of the oral cavity and jaws. Oral Surg. Oral Med. Oral Pathol. Oral Radiol. Endod..

[3] Singh U.R.G., Parkash J., Kumar P., Nath G., God S.C. (2003). Clinicoimmunologic study of plasmacytoma. Indian J. Allergy Asthma Immunol..

[4] Barros T.P., Savilha F.M., Amantea D.V., Campolongo G.D., Neto L.B., Alve N N. (2011). Plasmacytoma in the oral cavity: a case report. Int. J. Odontostomat..

[5] Algamra H., Alansar Z., Abdulhafez M., Taha R., Mahfouz A., Ibrahim F. (2011). Multiple myeloma presenting as unilateral proptosis: a case report. J. Clin. Exp. Ophthalmol..

[6] Poggio C. (2007). Plasmacytoma of the mandible associated with a dental implant failure: a clinical report. Clin. Oral Implants Res..

[7] Ozdemir R., Kayiran O., Oruc M., Karaaslan O., Kocer U., Ogun D. (2005). Plasmacytoma of the hard palate. J. Craniofac. Surg..

[8] Jeong J., Seo G., Song J., Park S. (2010). Solitary plasma cell myeloma on anterior maxilla: a case report. J. Korean Assoc. Maxillofac. Plast. Reconstr. Surgeons..

[9] Rodriguez-Caballero B., Sanchez-Santolino S., Gracia-Montesinos-Perea B., Gracia-Reija M.F., Gomez-Roman J., Saiz-Bustillo R. (2011). Mandibular solitary plasmacytoma of the jaw: a case report. J. Med. Oral. Pathol. Cir. Bucal..

[10] Agha R.A., Fowler A.J., Saetta A., Barai I., Rajmohan S., Orgill D.P., for the SCARE Group (2016). The SCARE Statement: consensus-based surgical case report guidelines. Int. J. Surg..

[11] Ariyarathenam A., Galvin N., Akoh J.A. (2013). Secondary extramedullary plasmacytoma causing small bowel intussusception in a patient with multiple myeloma - a case report. Int. J. Surg. Case Rep..

[12] Canger E.M., Celenk P., Alkan A., Gunhan O. (2007). Mandibular involvement of solitary plasmocytoma: a case report. Med. Oral Pathol. Oral Cir. Bucal..

[13] An S., An C., Choi K., Heo M. (2013). Multiple myeloma presenting as plasmacytoma of the jaws showing prominent bone formation during chemotherapy. Dentomaxillofacial Radiol..

[14] Singh A., Singh V., Sharma N. (2012). Solitary plasmacytoma of mandible: a rare case report. Int. J. Med. Dent. Sci..

[15] Souza L., Farias L., Santos L., Mesquita R., Martelli H., De-Paula A. (2007). Asymptomatic expansile lesion of the posterior mandible. Oral Surg. Oral Med. Oral Pathol. Oral Radiol. Endod..

[16] Daghighi M.H., Poureisa M., Shimia M., Mazaheri-Khamene R., Daghighi S. (2012). Extramedullary plasmacytoma presenting as a solitary mass in the intracranial posterior fossa. Iran. J. Radiol..

[17] Lesmes D., Laster Z. (2008). Plasmacytoma in the temporomandibular joint: a case report. Br. J. Oral Maxillofac. Surg..

[18] Di P. (2010). Micco. Solitary plasmacytoma of the jaw. J. Blood Med..

